# Early stages of building a rare disease registry, methods and 2010 data from the Belgian Neuromuscular Disease Registry (BNMDR)

**DOI:** 10.1007/s13760-014-0320-0

**Published:** 2014-06-24

**Authors:** Anna J. Roy, Peter Van den Bergh, Philip Van Damme, Kris Doggen, Viviane Van Casteren, The BNMDR Scientific Committee

**Affiliations:** 1 Health Services Research Unit, Department of Public Health and Surveillance, Scientific Institute of Public Health, Health Services Research, 14 rue Juliette Wytsman, 1150, Brussels, Belgium; 2 Neuromuscular Reference Centre, Cliniques Universitaires St-Luc, Brussels, Belgium; 3 Neuromuscular Reference Centre, University Hospital Gasthuisberg, Louvain, Belgium

**Keywords:** Neuromuscular, Diseases, Registry, Belgium, Prevalence

## Abstract

The Belgian Neuromuscular Disease Registry, commissioned in 2008, aims to collect data to improve knowledge on neuromuscular diseases and enhance quality health services for neuromuscular disease patients. This paper presents a clear outline of the strategy to launch a global national registry. All patients diagnosed with one of the predefined 62 neuromuscular disease groups and living in Belgium may be included in the yearly updated Registry. Basic core data is harvested through a newly designed web application by the six accredited neuromuscular reference centres. In 2010, 3,424 patients with a neuromuscular disorder were registered. The most prevalent disease group in the Registry is Hereditary Motor and Sensory Neuropathy, as similarly stated by other studies, albeit the prevalence in Belgium is five times lower: 6.5 per 100,000 in the north of Belgium, versus 17.0–41.0 per 100,000 in other areas of Europe. Very few patients were captured in the south of the country. With the aim to collect valuable epidemiological data, the registry targets to gather high quality data, that the sample to be representative of the population and that it be complete. The past 5 years of building the registry have improved its quality, albeit the consistent gap in data from the south of the country prevails, influencing the estimated prevalence of these diseases. To this day, the true burden of neuromuscular diseases in Belgium is not known but actions have been undertaken to address these issues.

## Introduction

On average, 6,000 rare diseases have been identified, affecting 6–8 % of the overall population [1]. In Europe, this would mean that there are 27–36 million people living with these isolated diseases. Tackling rare diseases is high on the European political agenda as stated in the 7th framework programme from 2007 to 2013 [1–3].

The term neuromuscular diseases encompasses conditions impacting the neuromuscular system, including the motor neurons, other spinal cord regions, the peripheral nerves, to the neuromuscular junction and the muscle. These diseases are rare, often hereditary, progressive in nature and without treatment. Some of these can be of autoimmune origin and treatable. The onset of symptoms ranges from childhood to adulthood and may vary from mild to more severe sensory and/or motor impairment, sometimes cardiac or respiratory involvement requiring life support and/or resulting in fatality. According to Orphanet, European portal for rare diseases and orphan drugs, the threshold of a rare disease is less than a person per 2,000 [4]. In contrast to the low frequency, neuromuscular diseases have a great impact on the quality of life of the patients, underlining the importance of a rapid diagnosis to guide the patient towards the most appropriate therapies.

According to the World Health Organisation, a patient registry is “a file of documents containing uniform information about individual persons, collected in a systematic and comprehensive way, in order to serve a pre-determined scientific, clinical or policy purpose” [5]. For the purpose of this article, a global registry was defined as a registry focusing on a group of related diseases. With this intention, the Belgian Neuromuscular Disease Registry (BNMDR) was created in 2008. Its goals are to increase the epidemiological knowledge of these diseases, to promote health services for patients having these diseases, to recruit patients for clinical trials and to provide information to the public health authorities for planning of health care in Belgium. It is a global registry collecting data on 62 different neuromuscular disease groups, highlighting the evolution of the diseases and monitoring their estimated prevalence throughout time in Belgium. To date, global neuromuscular disease registries are scarce, existing only in Canada [6], Australia [7], New Zealand [8], The Netherlands [9], and Belgium. However, most industrialised countries have certain rare disease-specific registries, independent of each other [10]. A global registry contrasts with disease-specific registries as it is time and resource saving in the preparation of tools and analysis of the data as the expertise is centralized. As each rare disease affects relatively small numbers of patients, grouping them enables further statistical analysis.

In this article we describe the methods used to build a rare disease registry and we carried out the first evaluation on the representativeness and exhaustiveness of the registry. For epidemiological purposes, the registry targets three aims: to collect data of high quality, have a representative sample and as ultimate goal to include the entire target population for completeness.

## Methods

### Organization and leadership

The registry began in 2008. Through agreements, it collects data by six Neuromuscular Reference Centres (NMRC) which is then analysed by the Scientific Institute of Public Health (WIV-ISP). It is funded by the National Insurance of Health and Disability Institute of Belgium (NIHDI) financing each NMRC per patient registered. A NMRC is a centre that has signed a convention with the NIHDI for health services for patients with neuromuscular diseases and provides a multi-disciplinary approach for those patients. There are two NMRCs in Brussels, three in Flanders, the northern part of Belgium, and one in Wallonia, the south. Patients, having been diagnosed with a neuromuscular disease, are seen in a NMRC of their choice, signing a yearly renewed convention. The leadership of the registry is carried out by the steering committee, composed of representatives from the NIHDI, WIV-ISP and experts from each NMRC. A national legal and consent framework was established to bind the partners in the project. Simultaneously a request for secondary use of data from patient health records was sent to the privacy commission of the sectorial health committee dealing with data protection issues and privacy enhancing techniques. The study protocol was accepted by the Commission d’Ethique Biomédicale Hospitalo-Facultaire of the Université Catholique de Louvain. After which, they contacted the five other medical ethics committees at the university hospitals harbouring each NMRC.

There is close collaboration with the eHealth platform, a Belgian public institution whose mission is to support patient information exchange between healthcare professionals. They ensure the respect of the privacy rights and patient confidentiality. Their aim is to optimize continuous care, patient security, simplify administrative steps and to offer support for health care politics [11]. The eHealth platform services also provide a third party coding to generate a unique identification number per patient based on the National Social Security Number, thus maintaining the patient’s anonymity.

The registry also gathers data for Translational Research in Europe for the Assessment and Treatment of Neuromuscular Diseases (TREAT-NMD), which is an European network providing the infrastructure and registry-building tools to professionals since 2007 [12]. The genetic mutation and detailed clinical data is gathered for Duchenne Muscular Dystrophy and Spinal Muscular Atrophy. In addition to facilitating the creation of registries, TREAT-NMD maintains an updated list of disease-specific registries Europe-wide for participants’ networking purposes.

### Study population

All patients followed in a NMRC, diagnosed with one of the determined 62 neuromuscular disease groups (Table 1) and living in Belgium can be included in the registry. Each patient has signed an informed consent form.

**Table 1:** Neuromuscular disease classification, BNMDR 2013

Muscular dystrophies		Disorder of the motor neurons	
1	Congenital muscular dystrophy	35	Amyotrophic lateral sclerosis
2	Duchenne muscular dystrophy	36	Primary muscular atrophy
3	Becker muscular dystrophy	37	Postpolio syndrome
4	Dystrophinopathy	38	Primary lateral sclerosis
5	Facioscapulohumeral dystrophy	39	Werdnig-Hoffman spinal muscular atrophy
6	Limb girdle muscular dystrophy	40	Intermediate spinal muscular atrophy
7	Emery-Dreifuss muscular dystrophy	41	Kugelberg–Welander spinal muscular atrophy
8	Distal myopathy	42	Adult spinal muscular atrophy
9	Oculopharyngeal muscular dystrophy	43	X-linked Bulbo-spinal muscular atrophy or Kennedy‘s disease
10	Myotonic dystrophy type 1	44	Distal spinal muscular atrophy
11	Myotonic dystrophy type 2	45	Hereditary spastic paraplegia
12	Other muscular dystrophies	46	Other disorders of motor neurons
Myotonic and relaxation disorders		Neuropathies	
13	Thomsen type myotonia congenita	Hereditary	
14	Becker type myotonia congenita	47	Hereditary motor and sensory neuropathy
15	Paramyotonia congenita	48	Hereditary neuropathy with liability to pressure palsies
16	Familial periodic paralysis	49	Hereditary sensory and autonomous neuropathy
17	Other myotonic disorders	Inflammatory	
Myopathies		50	Guillain-Barré syndrome
Congenital myopathies		51	Chronic inflammatory demyelinating polyneuropathy
18	Central core disease	52	Multifocal motor neuropathy
19	Multiminicore disease	53	Vasculitis
20	Nemaline myopathy	54	Neuropathy associated with paraproteinemia
21	Myotubular myopathy	55	Neuropathy associated with plasma cell dyscrasia
22	Centronuclear myopathy	56	Amyloidosis
23	Fibre type disproportion myopathy	57	Neuropathy in systemic disease
Metabolic myopathies		58	Other neuropathies
24	Muscle glycogenosis	Hereditary ataxias	
25	Disorders of fatty acid metabolism	59	Friedreich ataxia
26	Mitochondrial myopathy	60	Spinocerebellar ataxias
Inflammatory myopathies		61	Other Hereditary ataxias
27	Polymyositis	Various	
28	Dermatomyositis	62	Arthrogryposis multiplex congenita
29	Inclusion body myositis		
Other myopathies			
30	Other myopathies		
Disorder of the neuromuscular transmission			
31	Myasthenia gravis		
32	Congenital myasthenia		
33	Lambert-Eaton syndrome		
34	Other disorders of neuromuscular transmission		

### Data collection

The data collected originates from health records, gathered manually on a yearly basis by the centres. The basic core data collected covers socio-demographic information such as gender, age, and residence of the patients at district level, as well as the diagnosis and the evolution of their disease.

The diagnostic classification was recently updated by the experts for the 2010 data collection, altering the previous list of 47–62 neuromuscular disease groups. It was carried out to update the classification according to the research evolution (Table 1).

The 2010 cross-sectional data collection was carried out from 4 June 2011 to 14 January 2012. This article will focus on this data collection.

### Data collection tool

Throughout time, different tools were created for the data collection in the BNMDR. The first tool was an ACCESS-based application (version 0) which was installed locally on each computer of every NMRC. Due to local patient coding, this application did not permit to identify patients who had been seen at more than one centre, thus possibly including double entries in the database. It was also not conceived to ensure proper patient privacy.

The second tool was a web-based application (version 1), used for the 2010 data collection, accessible online with a secured code for the professional entering the data and equipped with central patient coding. To identify double entries, each patient was given a unique identifier code kept throughout time. This BNMDR identifier was defined by the services of the eHealth platform, functioning as a Trusted Third Party, by re-coding the unique National Social Security Number of the patient, thus allowing longitudinal research.

### Data management and plan of statistical analysis

After the data collection, error checking routines were carried out for range and consistency especially to identify in a unique way the double patient entries from patients having attended two different centres. Six of the 39 patients (*n* = 3,424) having been seen by two different centres had different diagnoses and were discarded. For the remaining 33 patients having a double entry, the first entry was retained as both diagnoses were the same.

Considering that the continuous variables were not normally distributed, the median was used, with confidence interval at 95 % (CI 95 %).

The prevalence was estimated and used as an indicator to evaluate the completeness of the registry knowing that it only represents an “estimated prevalence”. It was calculated as a quotient between the number of cases in the registry over the total population at the same time period, as a rate. The 95 % CI was calculated according to Poisson distribution. The total population living in Belgium, was calculated at mid-year, derived from an average based on the total population on the 1st of January 2010 and 1st of January 2011. Considering that Duchenne Muscular Dystrophy is X-linked, the denominator for that disease was based only on the male mid-population. For the estimated prevalence of the regions, the values used came from the 1st of January 2010, as there was no available data from 2011 [13, 14].

The descriptive statistical analysis was done with Stata 10.

## Results

The present study of 2010 was based on 3,424 patients with neuromuscular diseases. The overall registry population was composed of 56.1 % men and 43.9 % women. The median age was 46.0 years (CI 95 % 45.0–47.0 years). The median age of the first symptoms was known for 28.8 % of the patients and was 30.0 years (CI 95 % 25.0–33.0 years). The median age of diagnosis was known for 51.8 % of the patients and was 38.0 years old (CI 9 % 36.0–39.0 years). The time lapse between the first symptoms and diagnosis age was shown to be 2.1 years (CI 95 % 1.8–2.6) for 18.2 % of the patients (*n* = 622). The stage of disease was divided in four categories, 2.1 % were at the diagnosis stage where symptoms could not be observed, 69.3 % had symptoms but were still mobile, 26.0 % were wheelchair dependent and 2.6 % on life support. In 2010, 117 patients died, of those, 75 had amyotrophic lateral sclerosis.

**Fig. 1:**
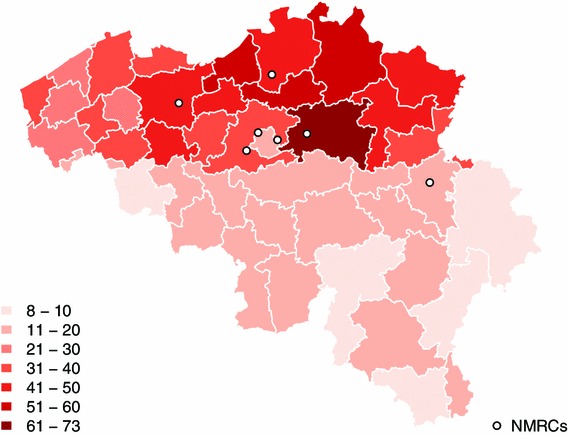
Estimated prevalence of the nine most prevalent neuromuscular disease groups per residential district per 100,000 inhabitants (*n* = 3,408) (BNMDR 2010)

An estimated prevalence of the nine most frequent diseases in BNMDR per 100,000 inhabitants can be found in Table 2. The prevalence of only three diseases in BNMDR are included in the literature prevalence range, Myotonic Dystrophy type 1, Spinocerebellar Ataxias, and Chronic Inflammatory Demyelinating Polyneuropathy whereas the other six have lower estimated prevalence compared to the literature values. Figure 1 is a map displaying the prevalence of the diseases per 100,000 inhabitants and by the 43 Belgian districts. Considerable differences can be noticed between the northern and the southern parts of the country. Tables 2 and 3 illustrate the distribution across region of four variables; gender, median age, stage of disease and frequency of the nine most prevalent diseases in the registry. The percentage of men per region is 60.2, 56.4, and 66.8 % for Brussels, Flanders and Wallonia, respectively. The median age is 47 for Brussels and Flanders but of 32 in Wallonia. In Brussels and Wallonia, almost three quarters of the patients are symptomatic whereas in Wallonia there are more than a third that are bound to a wheel-chair. Table 4 shows the ranking of the nine most prevalent diseases across regions. We notice variations according to region as highlighted by the color-codes to guide the reader.

**Table 2:** Prevalence of the nine most frequent neuromuscular diseases according to the literature review and the estimated prevalence in BNMDR per 100,000 inhabitants (BNMDR 2010)

	Prevalence in the literature	Overall prevalence in Belgium	Prevalence in the north of Belgium		Prevalence in the south of Belgium		
	Frequency	Frequency	CI^a^	Frequency	CI^a^	Frequency	CI^*^
HMSN [18–21]	17.0–41.0	4.2	3.8–4.6	6.5	5.8–7.1	1.3	1.0–1.7
DM1 [22, 23]	1.2–14.3	3.7	3.4–4.1	5.7	5.1–6.3	0.8	0.5–1.1
ALS [24, 25]	5.2–10.3	3.2	2.9–3.6	5.0	4.4–5.5	0.7	0.5–1.1
DMD [19, 26]^b^	16.7–28.6	4.3	3.8–4.9	5.1	4.4–6.0	0.3	0.3–0.4
LGMD [24, 27]	2.3	1.5	1.2–1.7	2.1	1.8–2.5	0.8	0.5–1.1
HSP [28]	1.6–18.5	1.3	1.1–1.5	2.1	1.7–2.5	1.4	0.5–3.3
SCA [29]	0.3–3.0	1.2	1.0–1.5	2.2	1.8–2.6	0	0
CIDP [30]	0.8–8.9	1.1	0.9–1.3	1.7	1.3–2.0	1.4	0.5–3.3
FSH [19]	10–20	0.9	1.0–1.5	1.2	1.0–1.5	0.5	0.3–0.8

^a^Confidence interval (CI), Poisson 95 %

^b^Only in the male population

**Table 3:** Gender, median age, stage of disease across region (BNMDR 2010)

	Brussels (*n* = 88)	Flanders (*n* = 1,813)	Wallonia (*n* = 211)
Gender (%)			
Males	60.2	56.4	66.8
Age (CI 95 %)			
Median	47 (30–50.1)	47 (45–48)	32 (26–38)
Stage of disease (%)			
Asymptomatic	0	1.2	1
Ambulatory	77.4	73.7	58.6
Wheel chair	20.2	22.8	35.3
Life support	2.4	2.2	5

**Table 4:** Disease frequency of nine more frequent disease groups across region (BNMDR 2010)

	Brussels (*n* = 88)			Flanders (*n* = 1,813)			Wallonia (*n* = 211)		
	Disease	Frequency	Percentage	Disease	Frequency	Percentage	Disease	Frequency	Percentage
2010	DM1	20	22.7	HMSN	404	22.3	DMD	58	27.5
	DMD	14	15.9	DM1	359	19.8	HMSN	46	21.8
	ALS	13	14.8	ALS	314	17.3	LGMD	27	12.8
	FSH	10	11.4	DMD	159	8.8	DM1	27	12.8
	HMSN	10	11.4	SCA	135	7.5	ALS	26	12.3
	CIDP	10	11.4	LGMD	132	7.3	FSH	17	8.1
	HSP	6	6.8	HSP	129	7.1	HSP	5	2.4
	LGMD	5	5.7	CIDP	104	5.7	CIDP	5	2.4
	SCA	0	0.0	FSH	77	4.3	SCA	0	0.0
		88	100		1,813	100		211	100

## Discussion

In this paper, we detailed how the Belgian global Neuromuscular Disease Registry was designed, then presented the most recent data collected, in 2010.

### Quality, representativeness and exhaustiveness of the data

The first goal of the registry is to collect high quality data. Different mechanisms were implemented during the data collection of the registry to improve its data quality. Constant interaction took place between the WIV-ISP and the data encoders of the NMRCs, minimizing data encoding errors. Drop down menus in the web-application were instated, to ensure uniform data classification. A unique BNMDR-id was generated per patient, which allows identification of each patient, eliminating double entries of patients visiting more than one centre. This identification number can also be used in the future for data comparison and internal validation of the data. Lastly, at the end of each data collection, a feedback report was sent to each centre, used as guidance for improvement of future data collections.

The second goal is that the sample captured in the registry be representative of the target population. Without gold standards guiding the test of this hypothesis, the distribution of four variables was examined. The four tested variables were gender, median age, stage of disease and ranking of the most prevalent disease across regions. In Table 3, differences across regions can be noted, such as a 10 % difference in percentage of males, found in the registry, between Flanders and Wallonia, or a 15 years difference in median age between both same regions. The stages of disease also differ across regions with more severely diseased patients in Wallonia. The ranking of the nine most prevalent diseases also differs across regions. As there is unequal distribution of patient characteristics across regions, with no valid epidemiological arguments supporting these findings, we can argue that these differences are related to poor representativeness of the target population.

The third goal of the registry is to include the entire target population. Table 2 charts the estimated prevalence in the registry in comparison to the values found in similar published research. The overall trend is lower values in the registry, for instance, five times lower for Hereditary Motor and Sensory Neuropathy and around three times lower for Myotonic Dystrophy type 1. Some of these differences can be explained by the wide range of origin of data used to calculate the prevalence in the literature, originating from various settings and using different classification systems. Another aspect which could show the exhaustiveness of the registry would be to observe the overall estimated prevalence of these diseases throughout the country. Figure 1 is a map representing the estimated prevalence of neuromuscular diseases per 100,000 inhabitants. We notice an under-registration of patients from the south of the country. Different hypotheses can explain this discrepancy. As a reminder, registration in the BNMDR only occurs through the neurologists in the NMRCs. It has been described that some patients coming to the NMRCs are not being included in the registry due to the burden of encoding on the neurologists who are seeing many patients but have no time for administration. Other patients are not coming to the NMRC, hence, are not being included in the registry. Hypotheses could be that, first, as neuromuscular diseases vary in intensity, some asymptomatic patients, or patients with mild symptoms, might not feel the need to consult a NMRC on a yearly basis, which are lost in the yearly data collections. Second, other patients might not come because there are no NMRCs in their area, especially in the south of the country. This could mean that patients are either seen by their general practitioners or in smaller hospitals not collecting data for the registry. Third, physical disability could be a barrier to reach the local NMRC. And last, some patients might not be aware that a registry exists. These prevalence and distribution discrepancies show that BNMDR has not reached its potential of having exhaustive data. For future epidemiological standards, we could investigate the possibility of expanding and reaching out to all patients with these diseases in the country.

## Strengths and limitations

Although the registry cannot claim at this point to be optimal, its setup process can be used as a guide for other countries ready to launch such registries. The transferable strengths of the registry include the coding, by a third party, of a unique and fixed BNMDR-identification number for each patient. This facilitates the consistent identification of double entries, thus improving the validity of the collected data and permitting future longitudinal research. In addition, this study has adapted the data collecting tool, shifting from a locally installed ACCESS tool, to an online web application allowing easier data transfer, data correction and data collecting.

Another strength is to begin with a minimum set of variables allowing the encoders to become familiar with the registry instrument before transforming it to a more complex instrument, ensuring better data entry. Finally, working in close collaboration with the partners, enabled improved data collection and resolving of potential issues that arose. The Dutch neuromuscular disease registry has already collected data from more than 10,000 patients in less than 10 years [9] and New Zealand around 100 after 5 years of careful planning [15]. Hence, Belgium, after 5 years of data collection, with data from 3,424 patients is within expected progression.

On the other hand, certain limitations influenced the quality of the data collection, the representativeness of the registry and its completeness. It is difficult to compare registries to one another as there is no international consensus as to which set of diseases should be considered in a neuromuscular disease registry, hence no gold standard. The non-standardised classification of diseases entails subjective classification and prevents international comparison. The experts of the BNMDR remodelled the earlier version of disease classification after the registry was launched, making comparison with previous data collections impossible. Also, the new classification should have mirrored the Orphanet classification, used in the Belgian central registry for rare diseases, with which it will soon merge. At last, the estimated prevalence is calculated on very small patient populations nationwide which may lead to biases.

## Opportunities for further research

The BNMDR has been operating for a few years and is now ready to expand from a basic set of core data to more detailed modules. The 2011 and 2012 latest data collection tool, called version 2, will include two extra modules, a scale measuring the ability to carry out daily activities called ACTIVLIM [16], and data collection for potential clinical trial selection, following the guidance of TREAT-NMD [17] protocol. ACTIVLIM is a self-administered, 22 closed multiple choice questionnaire that BNMDR patients are asked to complete on a yearly basis. It is based on Rasch analysis and can show evolution of disease throughout time. To simplify and ensure more quality in the data collection, an eHealth platform service module, “ConsultRN”, will be included in the tool. This will enable the web application to directly retrieve demographic data from the national registry into the BNMDR registry improving accuracy. The registry may also benefit from future efforts to extract diagnostic and treatment data directly from the patients’ electronic health records, thus reducing the registration burden on care providers. As the BNMDR aims to provide a tool to improve health care quality for patients with neuromuscular diseases, new variables will be added in the future data collections to benchmark these trends based on the principle of audit and feedback. The current feedback focuses mostly on improving the completeness and data quality of the registered patients and not clinical performance.

## Conclusions

This study shows that BNMDR can be seen as a pacesetter in guiding other countries to launch a global neuromuscular disease registry. We have seen how challenging it is to gather high quality data, for the sample to be representative and for the registry to be complete. To this day, the true burden of neuromuscular diseases in Belgium is not known. The past 5 years of building the registry have improved its quality, albeit the consistent gap in data from the south of the country prevents having a clear representation of the estimated prevalence of these diseases in Belgium. The results show an unequal planning of healthcare services for NMD patients in Belgium, an issue to be brought forward to the Public Health authorities. Today, patients are being recruited for clinical trials through the registry. It remains crucial, for comparison, to emphasise the need of an international classification, for all neuromuscular diseases such as the one being developed by the European network Orphanet.
